# Terahertz photon to dc current conversion via magnetic excitations of multiferroics

**DOI:** 10.1038/s41467-024-49056-9

**Published:** 2024-06-06

**Authors:** Makiko Ogino, Yoshihiro Okamura, Kosuke Fujiwara, Takahiro Morimoto, Naoto Nagaosa, Yoshio Kaneko, Yoshinori Tokura, Youtarou Takahashi

**Affiliations:** 1 Department of Applied Physics and Quantum Phase Electronics Center, University of Tokyo, Tokyo, Japan; 2 RIKEN Center for Emergent Matter Science (CEMS), Wako, Japan; 3 Tokyo College, University of Tokyo, Tokyo, Japan

**Keywords:** Ferroelectrics and multiferroics, Terahertz optics

## Abstract

Direct conversion from terahertz photon to charge current is a key phenomenon for terahertz photonics. Quantum geometrical description of optical processes in crystalline solids predicts existence of field-unbiased dc photocurrent arising from terahertz-light generation of magnetic excitations in multiferroics, potentially leading to fast and energy-efficient terahertz devices. Here, we demonstrate the dc charge current generation from terahertz magnetic excitations in multiferroic perovskite manganites with spin-driven ferroelectricity, while keeping an insulating state with no free carrier. It is also revealed that electromagnon, which ranges sub-terahertz to 2 THz, as well as antiferromagnetic resonance shows the giant conversion efficiency. Polar asymmetry induced by the cycloidal spin order gives rise to this terahertz-photon-induced dc photocurrent, and no external magnetic and electric bias field are required for this conversion process. The observed phenomena are beyond the conventional photovoltaics in semi-classical regime and demonstrate the essential role of quantum geometrical aspect in low-energy optical processes. Our finding establishes a paradigm of terahertz photovoltaic phenomena, paving a way for terahertz photonic devices and energy harvesting.

## Abstract (web summary)

Authors discover that the spin excitations of multiferroics convert the terahertz photon to the unidirectional charge current through the quantum geometrical aspect of optical process, paving the way to the terahertz photonics.

## Introduction

One ultimate goal of photonics is utilizing photon in all energy region, which is required for high-speed and quantum communications, long-range sensing technology as well as for energy conversion and harvesting. Usual photovoltaic response of, e.g., single-phase crystals, semiconductor devices, and biological molecules is, however, limited above the mid-infrared region, because the lower bound of available photon energy is determined by the charge gap of electronic states^1^. On the other hand, many statistic and dynamical phenomena in condensed matter are often dominated by low-energy elementary excitations, such as phonons and magnons^2^. It has long been believed that the photo-creation of such elementary excitations that never produce free electrons/holes cannot exhibit the dc photovoltaic response. Beyond this semi-classical consideration, recently established quantum geometrical nature of bulk photovoltaic effect (BPVE) suggests that a photo-creation process of elementary excitations can be accompanied by the dc charge current generation through their interaction with electrons even when the photon energy is far below the electronic band gap^3–7^; Terahertz photon with an energy of few meV is converted to the dc current through this process. This terahertz functionality cannot be achieved by the conventional BPVE with use of interband transitions with typical gap energy of eV. Furthermore, these theories imply that, if such elementary excitations are strongly coupled with the electronic states, the terahertz photon to dc current conversion efficiency can exceed that for the direct electron-hole pair creation.

Magnetoelectric multiferroics offer a promising avenue for the terahertz BPVE by using magnetic excitations strongly coupled with the electronic states. The basic concept of contemporary multiferroics is parity breaking by long-range spin orders, resulting in the magnetically induced ferroelectricity and chirality^8–10^. In multiferroics, the composite states of magnetic and ferroelectric orders exhibit versatile magnetoelectric phenomena beyond conventional ferroic materials^11,12^. To name a few, several types of nontrivial magnetoelectric domain switching have recently been demonstrated in multiferroics^13–15^. In the optical regime, the multiferroics host the electromagnon in terahertz region, which is a collective spin excitation with electric transition dipole^16–18^. The resonantly enhanced nonreciprocal optical effects of the electromagnon are a direct consequence of giant dynamical magnetoelectric coupling, manifesting that the spin dynamics is accompanied by the charge fluctuation^18–20^. Thus, the magnetic excitations of multiferroics are always coupled to the charge degrees of freedom, and hence are expected to lead to the realization of terahertz BPVE.

We have demonstrated the terahertz BPVE in multiferroics of spin origin. Our model multiferroic, perovskite manganite Eu_0.55_Y_0.45_MnO_3_ (EYMO), exhibits the spin-driven ferroelectricity in cycloidal spin spiral phase as well as the terahertz magnetic excitations^18,21–23^. It is revealed in this study that photo-creation of electromagnon and antiferromagnetic resonance (AFMR) gives rise to the dc charge current in the insulating multiferroic. The BPVE of electromagnon, which covers from sub-terahertz to 2 THz range, is found to exhibit giant conversion efficiency. The extended shift current mechanism in multiferroics, which incorporates the magnetic excitations coupled with the spin-driven ferroelectricity, reasonably explains the observed terahertz photocurrent.

## Results

We begin with the basic notion of terahertz BPVE in multiferroics. The shift current is originally established as one microscopic mechanism of BPVE for optical excitation above the band gap; zero-bias photocurrent is produced by the shift of the electronic wave packet in real space upon the optical transitions in the noncentrosymmetric crystal^3,4,24,25^. This real space shift is quantified by a geometrical quantity called shift vector, which involves difference of the Berry connections for valence and conduction bands associated with the optical transition. In this regard, the shift current mechanism for BPVE is a geometrical phenomenon that is closely related with the modern theory of electric polarization where the electronic part of spontaneous polarization (*P*) is expressed by the Berry phase of the Bloch wave functions constituting the electronic band structure^26^. In addition to the conventional ferroelectrics, the large photocurrent generation in mid-infrared region for polar Weyl semimetal TaAs is explained by the shift current mechanism^27^. The shift current can be extended for other real excitations such as low-energy elementary excitations by including the appropriate coupling with the electronic system; a photo-creation of an elementary excitation that accompanies electric polarization can in principle lead to the dc charge current^6,7,28^. The multiferroics of spin origin have the strong coupling between spin ordering and electronic system, so that the creation of terahertz spin excitations alters the electronic states and induces local electric polarization, giving rise to the BPVE.

**Fig. 1:**
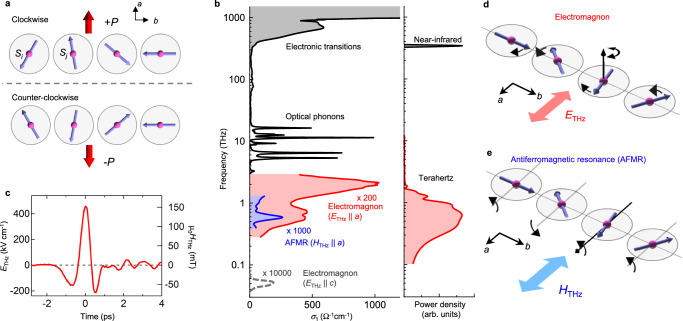
Terahertz spin excitations for multiferroic EYMO with cycloidal spin spiral. **a** Spontaneous *P* driven by cycloidal spin spiral. Opposite helicities of spin spiral with opposite *P* directions are illustrated. **b** Optical conductivity spectra of EYMO^30^; *E*||*a* for above 3 THz and the electromagnon (red), and *E*||*c, H*||*a* for the antiferromagnetic resonance (AFMR, blue) and the lower-lying electromagnon (dashed line) estimated from the former study^18^. (See Supplementary Fig. 2 for the derivation of terahertz spectra.) Power spectra of excitation terahertz and near-infrared pulses (right panel). **c** Time evolution of electric field (left axis) and magnetic field (right axis) of terahertz pulse. The power spectrum of (**c**) is plotted in (**b**). **d** Spin dynamics of electromagnon with ${E}_{{THz}}{||a}$. The neighboring spin oscillations are in opposite phase, and their rotation axis of spins is perpendicular to the spin spiral plane (upward arrow). **e** Spin dynamics of AFMR with ${H}_{{THz}}{||a}$. The rotation axis of spin is parallel to the *a* axis. (see Supplementary Fig. 3 for more detail of the lower-lying electromagnon).

**Fig. 2:**
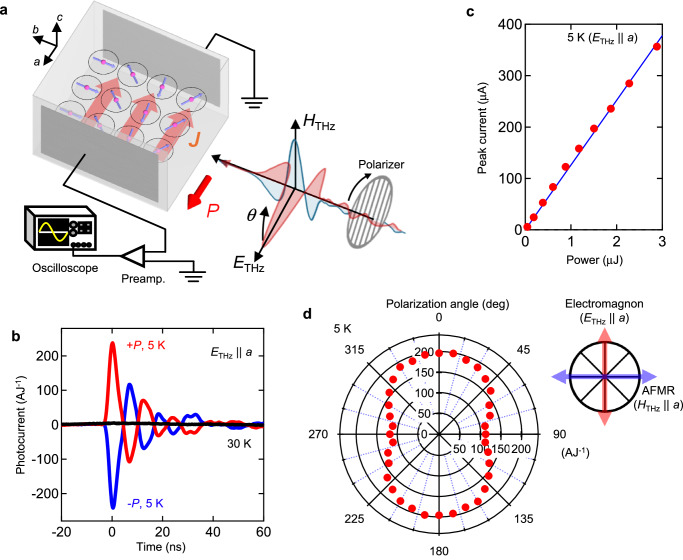
Observation of terahertz BPVE. **a** Schematics of experimental setup and of *ab*-plane spin spiral. Terahertz light-polarization is rotated by rotating the wire grid polarizer. The photocurrent flowing along the *a* axis is measured by the oscilloscope. **b** Time profile of photocurrent generated by terahertz pulses at the ferroelectric phase with $\pm P$ (5 K) and at the paraelectric phase (30 K). **c** Terahertz power dependence of photocurrent. Amplitude of photocurrent is plotted (see (**b**)). The blue line is a linear fit. **d** Terahertz light-polarization dependence of photocurrent flowing along the *a* axis at 5 K. The signal at 0° and 180° represents the photocurrent arising from electromagnon, while the signal at 90° and 270° represents the photocurrent arising from AFMR (see right upper panel). The amplitude of photocurrent is normalized by the energy of light inside the sample by considering the reflection loss at the sample surface.

In perovskite manganite EYMO, the *ab*-plane cycloidal spin spiral with spiral axis along the *b* axis (Fig. 1a) emerges below *T*_C1_ ~ 22 K and breaks the space inversion symmetry, giving rise to the spontaneous polarization (*P*) along the *a* axis^22^. This spin-driven *P* is described by the product of the vector spin chirality composed of neighboring spins at sites *i* and *j*, ${S}_{i}\times {S}_{j}$, and the unit vector connecting these sites, ${e}_{{ij}}$, as $P\propto \,{e}_{{ij}}\times ({S}_{i}\times {S}_{j})$^29^. Accordingly, the sign of helicity of spin cycloid, clockwise or counter-clockwise, is rigidly connected to the sign of *P* (Fig. 1a). This noncolinear spin ordering induces the electromagnon with electric transition dipole along the *a* axis (${E}_{{THz}}{||a}$, Fig. 1b); intrinsic broad spectral width with two peak structures, which covers from sub-terahertz to 2 THz, is generally observed for multiferroic perovskite manganites^16,18,30^. This electromagnon is ascribed to the phason of spin spiral at the Brillouin zone-edge (Fig. 1d)^31^; the GdFeO_3_-type lattice distortion folds the magnon at zone-edge to the zone center. The conventional AFMR with magnetic transition dipole along the *a* axis (${H}_{{THz}}{||a}$) emerges at 0.6 THz (Fig. 1b, e). (Note here that the electronic excitation energy or the band gap is observed only above 200 THz (Fig. 1b) far above these magnetic excitation energies.) We use the pulse terahertz-field generated by using differential frequency generation in LiNbO_3_ crystal (Fig. 1c)^32^. The power spectra of excitation terahertz pulses overlap with the resonant frequency of both spin excitations. Here, both electric (${E}_{{THz}}$) and magnetic (${H}_{{THz}}$) fields of terahertz pulses are exploited to create these magnetic excitations. The electromagnon (${E}_{{THz}}{||a}$) and AFMR (${H}_{{THz}}{||a}$) can be selectively excited by rotating the light-polarization for the *ac*-plane sample (Fig. 2a). The photocurrent synchronized to the terahertz pulses is detected by an oscilloscope (see methods).

Figure 2 summarizes the observation of terahertz photocurrent at the lowest temperature (5 K). In the *ab*-plane cycloidal phase with *P* | |*a*, the resonant excitation of electromagnon generates the photocurrent flowing along the *a* axis (5 K, Fig. 2a, b). In contrast, no photocurrent is observed in the centrosymmetric paraelectric phase (30 K), while the spectral weight of electromagnon remains even at 30 K (Supplementary Fig. 2). This clear correlation with the inversion symmetry breaking by spin ordering indicates that the observed terahertz photocurrent is caused by the BPVE, while a possible extrinsic origin, such as coming from electrodes, is excluded. Note that the oscillatory signal following the main peak at 20 ns is not essential but caused by the ringing in electrical circuit for current detection. In general, the photovoltaic effect is classified to the second order nonlinear optical effect; photocurrent flowing along *i* axis (${J}_{i}$) is expressed by the nonlinear conductivity tensor (${\sigma }_{{ijk}}^{E(2)}(\omega )$) as ${J}_{i}={\sigma }_{{ijk}}^{E\left(2\right)}\left(\omega \right){E}_{j}{E}_{k}$, where ${E}_{i}$ is an electric field of light, and *i*, *j*, and *k* are cartesian coordinates^7^. Here, ${\sigma }_{{aaa}}^{E\left(2\right)}\left(\omega \right)$ represents the BPVE for electromagnon with ${E}_{{THz}}{||a}$ (Fig. 2a). Since the sign of ${\sigma }_{{ijk}}^{\left(2\right)}\left(\omega \right)$ is determined by that of *P*, the sign reversal of terahertz photocurrent for the reversal of *P* is also consistent with the BPVE (Fig. 2b). The excitation power dependence in Fig. 2c clearly demonstrates that the photocurrent obeys the power law of photovoltaic effect, i.e., proportional to the photon number or equivalently the power flux of light being proportional to ${({E}_{{THz}})}^{2}$.

We examined the terahertz BPVE arising from the AFMR responding to ${H}_{{THz}}$ by rotating the light-polarization (Fig. 2a). Note that we always measure the current flowing along the *a* axis. A polar plot of photocurrent (Fig. 2d) shows the twofold anisotropic shape as expected for the *ac* surface of orthorhombic crystal. In addition to the electromagnon ($\theta=0^{\circ} \;\;{{{{{\rm{and}}}}}}\;180^{\circ}$ ), the substantial amplitude of photocurrent is observed for $\theta=90^{\circ} \,{{{{{\rm{and}}}}}}\,270^{\circ}$, indicating that the AFMR also exhibits the BPVE. Although only the electric field responses have been assumed for the BPVE so far, this photocurrent from AFMR demonstrates that the charge current generation driven by the magnetic field of light (${H}_{{THz}}$). Similar to the BPVE induced by ${E}_{i}$, we can express the nonlinear conductivity for the magnetic field of light (${\sigma }_{{ijk}}^{H\left(2\right)}$) as ${J}_{i}={\sigma }_{{ijk}}^{H\left(2\right)}\left(\omega \right){H}_{j}{H}_{k}$. The magnetic point group of *mm*21’ for cycloidal spin spiral allows the current generation by ${\sigma }_{{aaa}}^{H(2)}(\omega )$, being consistent with our observation for AFMR with ${H}_{{THz}}{||a}$. Therefore, in multiferroics, both ${E}_{{THz}}$ and ${H}_{{THz}}$ can be exploited for the conversion of terahertz photon into charge current through the creation of spin excitations. Note that the lower-lying electromagnon (dashed line in Fig. 1b and Supplementary Fig. 3), which is a Nambu-Goldstone mode of cycloidal spin order^17^, might be involved in the photocurrent generation through mode-mode coupling with AFMR^18^, although its center frequency (~60 GHz) is outside of the excitation pulse frequency in the present experiment.

To verify the above observations, we performed the theoretical calculation of nonlinear conductivity based on the extended shift current mechanism for multiferroics, where we employed a spin model that accounts for the spin structure for EYMO (see Supplementary Note 1 for more detail). The nonlinear optical conductivities derived from this theory show good agreement with the experimental values. It is also found that the AFMR exhibits the resonance of nonlinear optical conductivity, manifesting the response to the ${H}_{{THz}}$ (AFMR) gives rise to the terahertz BPVE in addition to that to the ${E}_{{THz}}$ (electromagnon). Thus, the theoretical model reproduces the polarization dependence of photocurrent from the AFMR excited with *c*-polarized light and the electromagnon excited with *a*-polarized light, along with a relatively lower conversion efficiency for AFMR as compared to the electromagnon. These results are consistent with the experimental observation in Fig. 2d.

**Fig. 3:**
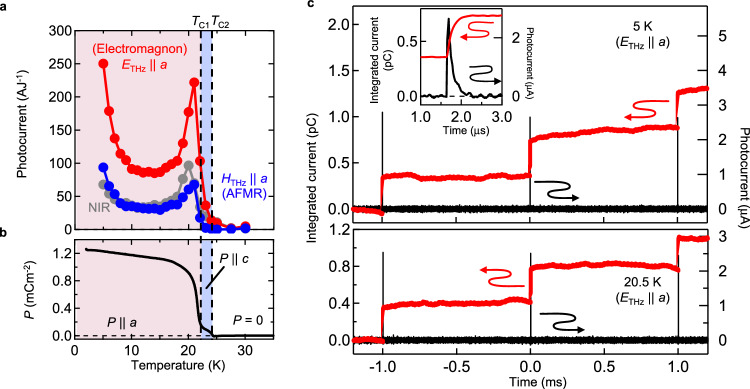
Temperature dependence and time integration of photocurrent. **a** Temperature dependence of amplitude of photocurrent efficiency for electromagnon (${E}_{{THz}}{||a}$, red), AFMR (${H}_{{THz}}{||a}$, blue) and near-infrared (NIR) (${E||a}$, gray). (See “Methods” section for the experimental conditions). **b** Temperature dependence of *P*. The *ab*-plane spin spiral phase with *P*||*a* is shaded in red, and the *bc*-plane spin spiral phase with *P*||*c* is shaded in blue. **c** The photocurrent for electromagnon (black) and its time integration (red) for pulse energy of 1 μJ at 5 K and 20.5 K. The repetition rate of terahertz pulse is 1 kHz and the terahertz pulses are irradiated at −1, 0, and 1 ms. The data around 1 μs is magnified (inset).

Figure 3a, b show the correlation between the photocurrent for spin excitations, that for electronic transition in near-infrared region (Fig. 1b, 800 nm), and *P*. Both photocurrents (red and blue lines) disappear in paraelectric phase with *P* = 0 above 24 K. Note that although EYMO shows the intermediate *bc*-plane spin spiral phase (*P* | |*c*) in a narrow temperature range between 22 K and 24 K (blue shaded area), a little photocurrent flowing along the *a* axis is allowed owing to the residual *P*||*a* (Fig. 3b). Beside the correlation with the transition temperature, we observed significant enhancement of photocurrent around the lowest temperature (5 K) as well as near the transition temperature (21 K), whereas *P* shows the monotonic decrease with increasing temperature. It should be noted that the spectral intensities of electromagnon and AFMR show the monotonic decrease as the temperature is increased (Supplementary Fig. 2). We examine the time integration of electromagnon photocurrent over a few pulses at 5 K and 20.5 K (Fig. 3c). At both temperatures, the step-like increase of integrated photocurrent is observed upon the irradiation of terahertz pulses every 1 ms, and the integrated current shows no reduction until the next pulse. These results conclusively manifest that the observed photocurrent arises from the genuine BPVE generating the steady-state photocurrent or equivalently the unidirectional current. The possible other effects such as photothermal-pyroelectric and -piezoelectric effects, which are always ac current accompanied by the back flow and never produce a net charge in present repetitive experiment, are excluded even at the peak near the transition temperature (Fig. 3a). (see Supplementary Note 3 for the heating effects by photo-irradiation.) We emphasize that the monotonic increase of charge in Fig. 3c does not indicate the gradual change of static polarization but demonstrates the creation of unidirectional current through the nonlinear optical effect, BPVE. Note that the radiative decay of magnetic excitations induces the back flow of shift current, while this effect should be insignificant: the radiative decay rate is proportional to ω^2^ if the oscillator strength is constant, so that the back flow being proportional to the radiative decay rate is substantially suppressed for the terahertz excitations as compared to the visible and near-infrared region.

The free energy analysis demonstrates the critical behavior, i.e., divergence, of second-order nonlinear susceptibility in zero-frequency limit, near the ferroelectric transition temperature, and phenomenologically explains the observed peak near around 21 K (see Supplementary Note 2). We also demonstrate the terahertz BPVE arising from electromagnon excitation in TbMnO_3_ with *bc*-plane cycloidal spin spiral (Supplementary Fig. 4). The photocurrent with similar nonmonotonous temperature dependence is observed in TbMnO_3_ as well, while the amplitude of photocurrent and their temperature anomalies are reduced. On the other hand, the enhancement of photocurrent efficiency is also observed at the lowest temperature. This low-temperature enhancement implies that the thermally excited magnon and phonon tend to suppress the conversion process in multiferroics, given that other degrees of freedom are frozen in the insulator. However, details of such mechanism cannot be specified at this stage and remain a future problem.

The BPVE for near-infrared light is also observed with similar temperature anomaly (NIR, Fig. 3a). Surprisingly, the terahertz spin excitations exhibit even larger photocurrent than this direct electronic transition above the band gap; the amplitude of photocurrent for electromagnon is 3.5 times as large as that for electronic transition at the lowest temperature. This result clearly indicates that the BPVE for terahertz spin excitations is dramatically enhanced in the multiferroics of spin origin.

## Discussion

**Fig. 4:**
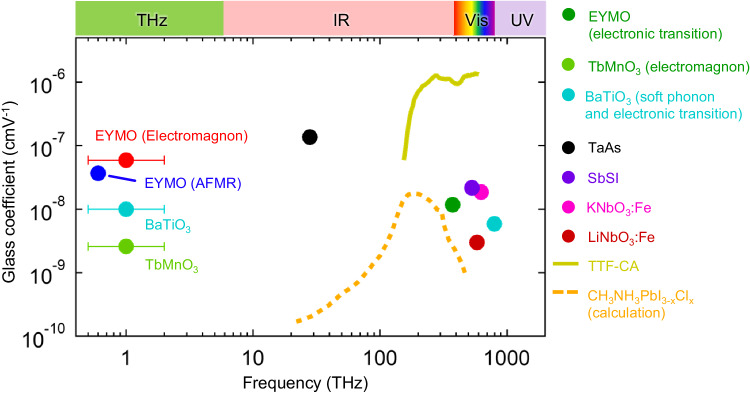
Comparison of photocurrent generation efficiency. The Glass coefficient in multiferroics; electromagnon (red), AFMR (blue), and electronic transition (dark green) in EYMO, electromagnon in TbMnO_3_ (light green, Supplementary Fig. 4). The error bars for the electromagnons (EYMO and TbMnO_3_) and for the phonon (BaTiO_3_) represent the bandwidth of the excitation terahertz pulse of the corresponding experiments. The Glass coefficient in various polar and ferroelectric materials are obtained from refs. ^27,28,37–42^.

Having provided substantial evidence for the giant terahertz BPVE for spin excitations in multiferroics, we compare it with the electronic transitions in various polar materials (Fig. 4). The Glass coefficient represents the conversion efficiency from absorbed power of light to the charge current (see Methods). The electromagnon exhibits *G* = 6 × 10^−8 ^cm V^−1^. This value is comparable with the high *G* = 16.5 × 10^−8 ^cm V^−1^ for mid-infrared electronic transition above 20 THz in Weyl semimetal TaAs, and larger than various ferroelectrics for visible-region excitations. It should be noted that the magnitude of *P* in multiferroics of spin origin (1.2 mC m^−2^ for EYMO, Fig. 3b) is usually smaller than that of the conventional ferroelectrics, for example *P* > 100 mC m^−2^ for BaTiO_3_^28^, so that the electrical or optical functionality arising from the symmetry breaking has been believed to be small. However, the observed giant *G* indicates the terahertz BPVE is less affected by the magnitude of *P*, and many multiferroics discovered in these two decades can be utilized for BPVE. By virtue of the BPVE for the various spin excitations in multiferroics, the frequency range applicable for photocurrent generation can be expanded from 10 GHz to 3 THz^18,30,33^. The spin-driven ferroelectricity with electromagnon realized at room temperature potentially leads to the terahertz and sub-terahertz photonic devices^34–36^.

The observed terahertz BPVE in multiferroics is reasonably explained by the extended shift current mechanism that describes the conversion from terahertz photon to dc charge current without electron-hole pair creation^6,7^. Since the shift current is generated on the time scale of the optical transition, the response time, in principle, can be shorter than a few pico seconds, while that in the present study is limited to 5 ns due to the electric circuit for current detection (Fig. 2b). The fast response expected for shift current could overcome the limit of response time for thermal detection of terahertz light. The microwave spintronic devices using the heavy metals and the ferroelectrics having high terahertz dielectric constant have appreciable loss in coupling with light propagating free space due to the high reflectivity. The same is true for semimetals. In contrast, the lower terahertz dielectric constant of multiferroics results in the efficient terahertz absorption by spin excitations, leading to the high conversion efficiency to dc charge current. The defect-tolerant characteristics of shift current^37^ further enhance the feasibility of terahertz devices based on multiferroics.

## Methods

### Sample preparation

Single crystals of Eu_0.55_Y_0.45_MnO_3_ and TbMnO_3_ were grown by the floating-zone method^21,22^. The dimensions of the crystals are typically 3 mm × 3 mm × 1 mm. The rectangular-shaped crystal was mounted in a cryostat with the electrodes for applying the dc electric field and measuring the photocurrent. To obtain the single domain of ferroelectric spin spiral order, the electric field was applied along the *a* axis and the *c* axis for Eu_0.55_Y_0.45_MnO_3_ (Fig. 2a) and TbMnO_3_, respectively, while cooling the sample down to the lowest temperature. The typical magnitude of electric field is 1.7 kV cm^−1^. No electric field was applied during the photocurrent measurement. In all measurements, no magnetic field was applied.

### Spontaneous polarization measurement

The electric polarization was deduced by measuring the pyroelectric current with increasing the temperature. The single ferroelectric domain state was obtained by the field cooling from ~100 K well above the magnetic-ordering temperature with application of the electric field of ±1.7 kV cm^−1^.

### Terahertz generation

For terahertz experiments, we used a regenerative amplified Ti:sapphire laser system with the pulse energy of 5 mJ, the pulse duration of 100 fs, the repetition rate of 1 kHz, and the center wavelength of 800 nm. The terahertz pulses were generated by the tilted-pulse front method with a LiNbO_3_ crystal^32^. The time waveform of the terahertz pulse was measured by the electro-optic (EO) sampling technique with using a ZnTe (110) crystal (Fig. 1c). To change the intensity and light-polarization of terahertz, two wire grid polarizers are used. The terahertz power is measured by using a terahertz power meter.

### Photocurrent measurement

We measured the pulse photocurrent signal synchronized to terahertz or near-infrared (800 nm) pulses by using a preamplifier and an oscilloscope. Spot size of both excitation light, terahertz and near-infrared, was about 1 mm and the spot was at the center of the sample, away from the metal contacts. The terahertz pulse energy of 0.9 μJ was used in Fig. 3a for both electromagnon and AFMR. To keep the similar excitation condition for NIR excitations, the pulse energy of 1 μJ was used for NIR excitations. The bandwidth of the preamplifier was 200 MHz for the scans in Figs. 2 and 3a and was 1 MHz for the scans over 2.5 ms in Fig. 3c. Accordingly, in Fig. 3c, the pulse width of the photocurrent is broadened, while the peak amplitude is decreased. To reduce the background signal, we measured the waveform with and without the incident light and calculated the difference between them. The amplitude of the photocurrent is normalized by the excitation energy inside the sample by considering the reflection loss at the sample surface.

### Optical conductivity spectra

The optical conductivity spectrum above 3 THz in Fig. 1b was obtained through the Kramers-Kronig analysis of the reflectivity spectra. We measured the reflectivity spectra with use of a Fourier-transform-type spectrometer in the infrared region above 3 THz, a monochromator-type spectrometer in the visible and ultraviolet regions below 4 eV (967 THz), and synchrotron radiation at UV-SOR, Institute for Molecular Science in the ultraviolet region above 4 eV.

### Evaluation of the Glass coefficient

The photocurrent response can be characterized by the Glass coefficient $G$. The photocurrent $J$ is described for the incident light normal to the sample surface by,

1 $$J=G{i}_{{{{{{\rm{abs}}}}}}}w,$$

where ${i}_{{{{{{\rm{abs}}}}}}}$ is the absorbed power density of light and $w$ is the width of the sample surface. Here the uniform and cw excitation are assumed. We estimate the terahertz Glass coefficient for the pulse photocurrent measurements; $G$ is calculated by $Q/w{i}_{{abs}}^{P}$, where $Q,$ $w$ and ${i}_{{abs}}^{P}$ represent the integrated photocurrent per pulse, the width of the photo-irradiated area and the excitation density per pulse, respectively.

### Supplementary information

Supplementary Information

Peer Review File

## Data Availability

Source data are provided with this paper. All other data that support the plots within this paper are available from the corresponding authors upon reasonable request. The data that support the plots of this study are available from the corresponding author upon reasonable request.
