# Supplementary material for: Terahertz photon to dc current conversion via magnetic excitations of multiferroics
Source: Nat Commun. 2024 Jun 6;15:4699. doi: 10.1038/s41467-024-49056-9 (PMC11156647; doi:10.1038/s41467-024-49056-9)
Supplement: Supplementary file 1 — Supplementary Information [file 41467_2024_49056_MOESM1_ESM.pdf]

Supplementary information for “Terahertz photon to dc current conversion via magnetic excitations of multiferroics”

Makiko Ogino[1], Yoshihiro Okamura[1], Kosuke Fujiwara[1], Takahiro Morimoto[1], Naoto Nagaosa[2] Yoshio Kaneko[2], Yoshinori Tokura[1,2,3] & Youtarou Takahashi[1,2]*

[1]Department of Applied Physics and Quantum Phase Electronics Center, University of Tokyo, Tokyo 113-8656, Japan

[2]RIKEN Center for Emergent Matter Science (CEMS), Wako 351-0198, Japan

[3]Tokyo College, University of Tokyo, Tokyo 113-8656, Japan

*Corresponding author. youtarou-takahashi@ap.t.u-tokyo.ac.jp

Supplementary Note 1: Theory for nonlinear optical responses of magnetic excitations

Model

We consider the cycloidal spin structure for the $J_1 - J_2$ spin chain. The spin Hamiltonian is given by

$$H = \sum_{\langle i.j \rangle_\alpha} \frac{J_\alpha}{2} \boldsymbol{S}_i \cdot \boldsymbol{S}_j + \sum_{\langle i.j \rangle_b} \frac{\boldsymbol{D}}{2} \left(\boldsymbol{S}_i \times \boldsymbol{S}_j \right) + \sum_{\langle\langle i.j \rangle\rangle_b} \frac{J_2}{2} \boldsymbol{S}_i \cdot \boldsymbol{S}_j. \tag{1}$$

Here, $\sum_{\langle i.j \rangle_\alpha}$ and $\sum_{\langle\langle i.j \rangle\rangle_b}$ represent the sum over the nearest neighbor and the next nearest neighbor sites along the α direction, where α runs a, b and c. For the nearest neighbor sites along the b axis, $\boldsymbol{D}$ is the Dzyaloshinskii-Moriya (DM) vector. We consider the case $J_b < 0,\ J_2 > J_b/4, J_c > 0, J_a < 0$ and $\boldsymbol{D} = (0,0,D)$. In this case, the ground state spin configuration is the ab plane cycloidal with a spiral axis along the b direction. The classical spin vector is written as

$$\boldsymbol{S}_i = S(\cos(\boldsymbol{Q} \cdot \boldsymbol{r}), \sin(\boldsymbol{Q} \cdot \boldsymbol{r}), 0) \tag{2}$$

where $\boldsymbol{Q} = (0, Q/d_b, \pi/d_c)$, $\boldsymbol{r}$ is the position of $\boldsymbol{S}_i$ and d_α is the lattice constant along the α direction.

Now, we consider the external field and the electric polarization. We apply the light propagating along the b direction. Thus, the electric field and the magnetic field is applied in the ac plane. In this model, the electric polarization along the a direction appears due to the inverse DM interaction and the exchange striction. Thus, the coupling of the external electric/magnetic fields along the a direction can be written as

$$H_{ext} = -E_a \sum_{\langle i,j \rangle_b} \left[(-1)^{i_b} \delta\Pi \boldsymbol{S}_i \cdot \boldsymbol{S}_j + \Pi \left(\boldsymbol{S}_i \times \boldsymbol{S}_j \right)_c \right] - B_a \sum_i g\mu S_i^a, \tag{3}$$

where $\delta\Pi$ and Π is the exchange and Dzyaloshinskii-Moriya interaction between nearest neighbor spins along the b direction induced by the electric field, respectively.
Note that E_a and B_a become nonzero for a-polarized and c-polarized light, respectively, and do not appear simultaneously. We adopted this notation for simplicity for the latter analyses.

Magnon Hamiltonian and nonlinear response

To obtain the magnon Hamiltonian, we rotate the spin quantization axis along the cycloidal spin structure as $\boldsymbol{S} = \mathcal{R}\, \tilde{\boldsymbol{S}}_i$and apply the Holstein-Primakoff transformation for $\tilde{\boldsymbol{S}}_i$. The Holstein-Primakoff transformation is [1]

$$\tilde{S}_i^+ = \hbar \sqrt{2S - a_i^\dagger a_i a_i} \sim \hbar\sqrt{2S} a_i, \tag{4}$$

$$\tilde{S}_i^- = \hbar a_i^\dagger \sqrt{2S - a_i^\dagger a_i} \sim \hbar\sqrt{2S} a_i^\dagger, \tag{5}$$

$$\tilde{S}_i^a = \hbar\left(S - a_i^\dagger a_i\right), \tag{6}$$

where $a_i^\dagger$ is a bosonic creation operator and $\tilde{S}_i^\pm = \tilde{S}_i^b \pm i\tilde{S}_i^c$. By using the Fourier transformation, $a_i = \frac{1}{\sqrt{N}}\sum_{\boldsymbol{k}} a_i\, e^{ik\cdot(r_i)}$, we obtain the magnon operator in k space. Within a linear approximation, the magnon Hamiltonian can be written as

$$H = \sum_{\boldsymbol{k}} \left[\Psi_{\boldsymbol{k}}^\dagger \left(H_{0,\boldsymbol{k}} - E_a\, \Pi_{\boldsymbol{k}} - B_a H_{\mathrm{m},\boldsymbol{k}} \Psi_{\boldsymbol{k}} \right) + \left(-E_a\, \mathrm{u}_{\mathrm{e},\boldsymbol{k}} - B_a \mathrm{u}_{\mathrm{m},\boldsymbol{k}} \right) \sqrt{N} \Psi_{\boldsymbol{k}} \right], \tag{7}$$

where $\Psi_{\boldsymbol{k}} = \left(a_{A,\boldsymbol{k}}, a_{B,\boldsymbol{k}}, a_{A,-\boldsymbol{k}}^\dagger, a_{B,-\boldsymbol{k}}^\dagger\right)^T$ with sublattice indices A and B, and N is the total number of A sites and B sites. Here, the sublattice degrees of freedom (A and B) are introduced in order to incorporate the asymmetry arising from the exchange striction expressed in equation (3). The unperturbed magnon Hamiltonian $H_{0,\boldsymbol{k}}$ is given by

$$H_{0,\boldsymbol{k}} = S \begin{pmatrix} A_{0,\boldsymbol{k}} & B_{0,\boldsymbol{k}} & C_{0,\boldsymbol{k}} & D_{0,\boldsymbol{k}} \\ B_{0,\boldsymbol{k}}^* & A_{0,\boldsymbol{k}} & D_{0,-\boldsymbol{k}} & C_{0,\boldsymbol{k}} \\ C_{0,-\boldsymbol{k}}^* & D_{0,-\boldsymbol{k}}^* & A_{0,-\boldsymbol{k}} & B_{0,-\boldsymbol{k}}^* \\ D_{0,\boldsymbol{k}}^* & C_{0,-\boldsymbol{k}}^* & B_{0,-\boldsymbol{k}} & A_{0,-\boldsymbol{k}} \end{pmatrix}, \tag{8}$$

with

$$\begin{aligned} A_{0,\boldsymbol{k}} = &-J_b \cos(Qd_b) + J_c - J_a(1 - \cos k_a d_a) \\ &+ J_2\left(\cos^2(2Qd_b)\cos(2k_b d_b) - \cos(2Qd_b)\right) - D\sin(Qd_b), \end{aligned} \tag{9}$$

$$\mathrm{B}_{0,\boldsymbol{k}} = J_b \cos^2\left(\frac{Qd_b}{2}\right)\cos(k_a d_b) + \frac{D}{2}\sin(Qd_b)\cos(k_a d_b), \tag{10}$$

$$\mathrm{C}_{0,\boldsymbol{k}} = -J_2 \sin^2(Qd_b)\cos(2k_b d_b) - J_c \cos k_c d_c\,, \tag{11}$$

$$\mathrm{D}_{0,\boldsymbol{k}} = -J_b \sin^2\left(\frac{Qd_b}{2}\right)\cos(k_b d_b) + \frac{D}{2}\sin(Qd_b)\cos(k_b d_b)\,. \tag{12}$$

The electric polarization along a direction is given by

$$\Pi_{\boldsymbol{k}} = S \begin{pmatrix} A_{e,\boldsymbol{k}} & B_{e,\boldsymbol{k}} & C_{e,\boldsymbol{k}} & D_{e,\boldsymbol{k}} \\ B_{e,\boldsymbol{k}}^* & A_{e,\boldsymbol{k}} & D_{e,-\boldsymbol{k}} & C_{e,\boldsymbol{k}} \\ C_{e,-\boldsymbol{k}}^* & D_{e,-\boldsymbol{k}}^* & A_{e,-\boldsymbol{k}} & B_{e,-\boldsymbol{k}}^* \\ D_{e,\boldsymbol{k}}^* & C_{e,-\boldsymbol{k}}^* & B_{e,-\boldsymbol{k}} & A_{e,-\boldsymbol{k}} \end{pmatrix}, \tag{13}$$

with

$$A_{e,\boldsymbol{k}} = \Pi \sin(Qd_b)\,, \tag{14}$$

$$B_{e,\boldsymbol{k}} = -\frac{\Pi}{2}\sin(Qd_b)\cos(k_b d_b)\,, \tag{15}$$

$$C_{e,\boldsymbol{k}} = 0, \tag{16}$$

$$D_{e,\boldsymbol{k}} = -\frac{\Pi}{2}\sin(Qd_b)\cos(k_b d_b)\,. \tag{17}$$

The single magnon coupling to the electric field is given by $-E_a u_{e,\boldsymbol{k}}$ with

$$u_{e,\boldsymbol{k}} = 2S\sqrt{\frac{S}{2}}\delta\Pi\sin(Qd_b)\,\delta(\boldsymbol{k}-\boldsymbol{0})(1,1,-1,-1). \tag{18}$$

The single magnon coupling to the magnetic field $u_{m,\boldsymbol{k}}$ is given by $-B_a u_{m,\boldsymbol{k}}$ with

$$u_{m,\boldsymbol{k}} = \mathrm{i}\frac{\sqrt{S}}{2}\mu\delta(\boldsymbol{k}-\boldsymbol{Q})(1,1,1,1) - \mathrm{i}\frac{\sqrt{S}}{2}\mu\delta(\boldsymbol{k}+\boldsymbol{Q})(1,1,1,1). \tag{19}$$

For the magnetic field, we only consider the single-magnon term because we focus on the nonlinear conductivity from the single-magnon resonance below, and neglect $H_{m,\boldsymbol{k}}$.

Now we study nonlinear electric current induced by the electric field and magnetic field. To look at the overall feature of nonlinear optical conductivity, the contribution from the single-magnon term ($u_{e,\boldsymbol{k}}$ and $u_{m,\boldsymbol{k}}$) is more important than the contribution from the two-magnon term, since the former gives sharp resonances in light frequency while the latter gives broad spectra. Here, we focus on the contribution from the single-magnon term. Namely, we study the single-magnon contribution to the nonlinear conductivities $\sigma(0,\omega,-\omega)_e^{aaa}$ and $\sigma(0,\omega,-\omega)_m^{aaa}$ which are defined as

$$J^a(0) = \sigma(0,\omega,-\omega)_{\mathrm{e}}^{aaa}E_a(\omega)E_a(-\omega), \tag{20}$$

$$J^a(0) = \sigma(0,\omega,-\omega)_m^{aaa}B_a(\omega)B_a(-\omega). \tag{21}$$

The formulas for the nonlinear conductivities are written as [2]

$$\sigma^{\mu\nu\nu}(0,\omega,-\omega)_\beta = -\frac{2\pi}{V_{\mathrm{cell}}\hbar}\sum_{\boldsymbol{k}}\sum_{\mathrm{a}}\left(\mathrm{u}_{\beta,\boldsymbol{k}}\mathrm{V}_{\boldsymbol{k}}\right)_{\mathrm{a}}(\mathrm{V}_{\boldsymbol{k}}^{-1}\sigma_3\Pi_{\boldsymbol{k}}\mathrm{V}_{\boldsymbol{k}})_{\mathrm{aa}}\left(\mathrm{V}_{\boldsymbol{k}}^{-1}\sigma_3\mathrm{u}_{\beta,\boldsymbol{k}}^{\dagger}\right)_{\mathrm{a}}$$

$$\times\left[\delta(\hbar\omega-\varepsilon_{\boldsymbol{k},\mathrm{a}})+\delta(-\hbar\omega-\varepsilon_{\boldsymbol{k},\mathrm{a}})\right] \tag{22}$$

Here, $V_{\boldsymbol{k}}$ is a matrix which diagonalizes $H_{0,\boldsymbol{k}}$ as $V_{\boldsymbol{k}}^{\dagger}\sigma^3 H_{0,\boldsymbol{k}}V_{\boldsymbol{k}} = \varepsilon_{\boldsymbol{k}}$, where $\varepsilon_{\boldsymbol{k}}$ is the magnon energy and $\sigma_3 = diag(1,1,-1,-1)$. u_β represents single magnon coupling to the external fields. β is the index which represents m or e.

Result

We show the magnon band dispersion and nonlinear conductivity in Supplementary Fig.1. The magnon dispersion is shown for $k_c d_c = 0$ and $k_c d_c = \pi$ in Supplementary Fig. 1(a) and Supplementary Fig. 1(b). The single-magnon coupling to the external fields gives rise to peak structures around the single-magnon resonances in the optical conductivity and the nonlinear conductivity. In particular, from Eq. (18), the electric field excites the magnon with $\boldsymbol{k} = \boldsymbol{0}$. Accordingly, $\sigma(0,\omega,-\omega)_e$ has a peak structure around $\omega = \varepsilon_{\boldsymbol{0}}$ (Supplementary Fig. 1(c)), which corresponds to electromagnon resonance represented by the blue arrow in Supplementary Fig. 1(a). On the other hand, as shown in Eq. (19), the magnetic field excites a magnon with $\boldsymbol{k} = \pm\boldsymbol{Q} = \pm(0, Q/d_b, \pi/d_c)$. Thus $\sigma(0,\omega,-\omega)_m$ has a peak structure around $\omega = \varepsilon_{\boldsymbol{Q}}$ (Supplementary Fig. 1(d)), which

corresponds to antiferromagnetic resonance represented by the red arrow in Supplementary Fig. 1(b).

Next, we estimate orders of magnitude of the nonlinear conductivity and the photocurrent. Given the polarization of the material $P \sim 1 \text{ mC m}^{-2}$ and the volume of the unit cell $V_{\text{cell}} \sim 10^2 \text{ Å}^3$, each bond with neighboring spins gives the electric polarization of $\frac{PV_{cell}}{S^2} \sim 1.6 \times 10^{-3}\ e\text{Å}$ with $S = 2$. To roughly reproduce this amount of $\frac{PV_{cell}}{S^2}$ by adding exchange striction contribution and inverse DM contribution to the polarization for each bond, we adopt the microscopic parameters regarding the electric polarization as $\Pi \sim 1 \times 10^{-3}\ e\text{Å}$ and $\delta\Pi \sim 6 \times 10^{-4}\ e\text{Å}$. These parameters lead to the nonlinear conductivity of $\sigma_e \sim 10 \text{ nA V}^{-2}$ using the peak value in Supplementary Fig. 1c. We can directly compare this theoretically obtained value of σ_e with the experimental value. When the penetration depth of irradiated light is enough shorter than the sample thickness, the nonlinear optical conductivity is given by

$$\sigma_e = \sigma_{aaa}^{(2)} = \frac{\alpha_a G_{aaa}}{2}\sqrt{\frac{\varepsilon_0}{\mu_0}},$$

where α_j and G_{aaa} are absorption coefficient and Glass coefficient (see main text and Method) for the electromagnon polarized along the a axis. From the penetration depth (~100 μm) and Glass coefficient ($6\times10^{-8}\ cm\,\text{V}^{-1}$, see main text), we obtain the experimental nonlinear optical conductivity as 8 nA V^{-2}. The experimental and theoretical σ_e show the quantitative agreement.

Furthermore, we can reproduce the polarization dependence of the photocurrent as follows. The ratio between σ_e and σ_m is given by

$$\frac{\sigma_e}{\sigma_m} = 10^2\left(\frac{\delta\Pi}{\mu}\right)^2 \sim 1 \times 10^{-16} \text{ m}^2\,\text{T}^2\text{V}^{-2} \tag{23}$$

Since the electric and magnetic fields of light is related with $E = cB$ with c being the speed of light, we obtain

$$\frac{\sigma_e E^2}{\sigma_m B^2} \simeq 10, \tag{24}$$

indicating that the magnitudes of the electric field-induced nonlinear current is estimated to be about 10 times larger than that of the magnetic field-induced nonlinear current with c-polarized light. This behavior is consistent with the polarization angle dependence of the observed photocurrent.

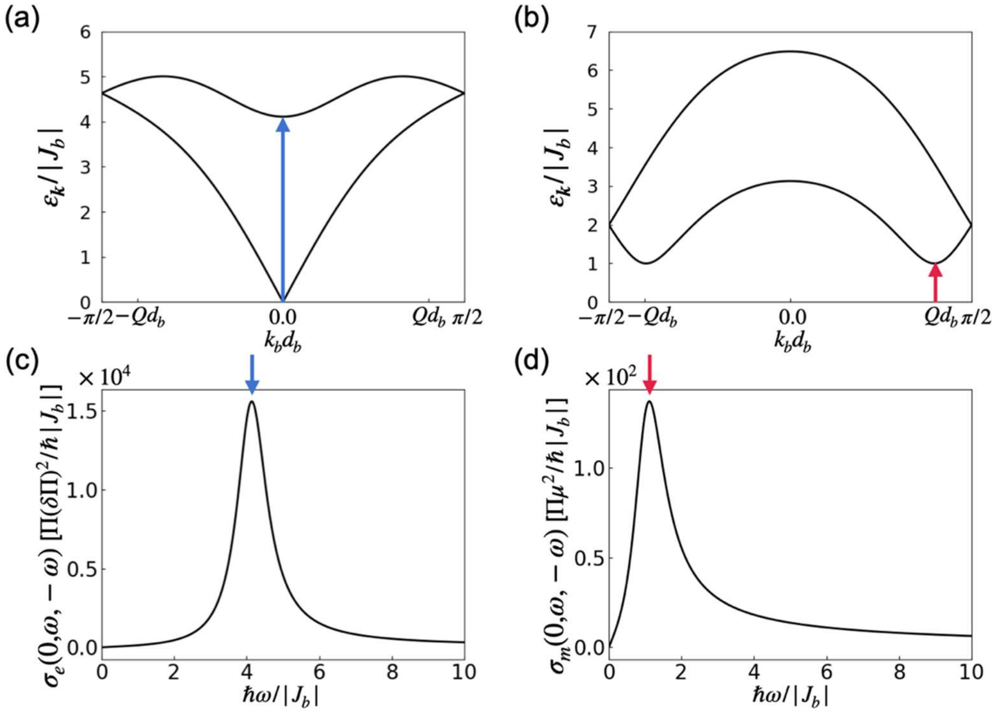

Supplementary Figure 1| Calculation of shift current for magnetic excitations. a, The magnon band dispersion with $k_c = 0$. **b**, The magnon band dispersion with $k_c d_c = \pi$. Arrows represent optical single-magnon resonance (blue for the electromagnon resonance excited by the electric field and red for the antiferromagnetic resonance excited by the magnetic field). **c**, Nonlinear conductivity σ_e. **d**, Nonlinear conductivity σ_m. We use the parameters; $J_b = -0.85$ meV, $J_2 = 0.68$ meV, $J_c = 1.28$ meV, (from Ref [3]) $J_a = -1.0$ meV, $D = -0.05$ meV, $\Pi = 1.0\ d_b$, $\delta\Pi = 0.1\ d_b$, $\mu = 1.0\ d_b$, $g = 2$, and $S = 2$. We adopted the energy broadening $\gamma = 0.5$ meV.

Supplementary Note 2: Free energy analysis for nonlinear susceptibility

We can phenomenologically deduce the temperature dependence of nonlinear susceptibility by a free energy analysis. We assume that the expansion of free energy F near the ferroelectric phase transition point as follows.

$$F = a(T)P^2 + \frac{1}{2}P^4 - E \cdot P \tag{25}$$

$$a(T) = A(T - T_C), \quad A > 0$$

Here A is an arbitrary constant. Below T_C, the net polarization is described with the spontaneous polarization P_0 and the series expansion of E as

$$P = P_0 + \alpha_1 E + \alpha_2 E^2 + O(E^3). \tag{26}$$

Hereafter, we consider the expansion up to second order of E. By minimizing F with respect to P, we obtain the stable condition.

$$\frac{\partial F}{\partial P} = 2a(T)P + 2P^3 - E = 0 \tag{27}$$

Substituting Eq. (26) into Eq. (27), we obtain the following equations for each order of E.

$$\begin{cases} a(T)P_0 + P_0^3 = 0 \\ a(T)\alpha_1 E + 3P_0^2\alpha_1 E = \frac{1}{2}E \\ a(T)\alpha_2 E^2 + 3P_0^2\alpha_2 E^2 + 3P_0\alpha_1^2 E^2 = 0 \end{cases} \tag{28}$$

Accordingly, the temperature dependence of P_0, α_1 and α_2 are derived as follows.

$$|P_0| = \sqrt{|a(T)|} \tag{29}$$

$$\alpha_1 = \frac{1}{4|a(T)|} \tag{30}$$

$$\alpha_2 = -\frac{3}{32}\frac{1}{|a(T)|^{5/2}} \tag{31}$$

Since $P = \chi_2(T)E^2$, the second order nonlinear susceptibility $\chi_2(T)$ equals to α_2.

$$\chi_2 = -\frac{3\mathrm{sign}(P_0)}{32}\frac{1}{|A(T-T_C)|^{5/2}} \tag{32}$$

The χ_2 shows the strong divergence near T_C from below, while it is 0 above T_C. The nonlinear optical conductivity $\sigma^{(2)}(\omega)$ expresses the dc current generation by ac E field, while χ_2 primarily describes a response to a static electric field. Hence, $\sigma^{(2)}(\omega)$ is also expected to show a similar temperature dependence as far as the frequency ω is not too high, i.e., lower than the exchange energy typically. Indeed, this should be the case since the power spectra of the incident THz light shows an overall deviation to lower frequencies from the peak value of the electromagnon spectrum (Fig. 1b). This result qualitatively explains the experimentally observed enhancement of photocurrent near T_{C1} (Fig. 3a). We note that the second order phase transition is assumed for the free energy analysis, while the actual phase transition to the ferroelectric cycloidal spin spiral is of the first order. Therefore, the critical behaviors are much reduced in the present case as experimentally observed.

Supplementary Note 3: Evaluation of heating effect by terahertz absorption

Absorption of terahertz photon results in the increase of temperature through the thermalization processes. Upper limit of temperature increase by pulse terahertz excitation can be estimated by heat capacity[4], penetration depth (~ 100 μm), spot size (~ 1 mm) and pulse energy (~ 1 μJ). For example, the estimated temperature rise per pulse is 0.06 K at the phase transition point (22 K). In this estimation, we omit the thermal diffusion in the sample and the heat conduction to the heat bath (cryostat), so that the actual temperature of the photo-induced heating effect is much smaller than this estimation. Clear correlation between the phase transition and photocurrent in Fig. 3a also demonstrates the negligible temperature increase by photo-irradiation.

In all experiment, we did not find obvious responses arising from the photo-thermal pyroelectric current that is observed as AC response in the repetitive experiments. There are two possible reasons. First one is that the photothermal dynamics is shorter than the time-resolution of this experiment (~200 ns, see inset to Fig. 3(c)). In this case, the AC photothermal current is canceled within the time resolution. Second one is that the thermal diffusion to the heat bath is more dominant than the heating in the crystal. Both effects may contribute to the actual experiments.

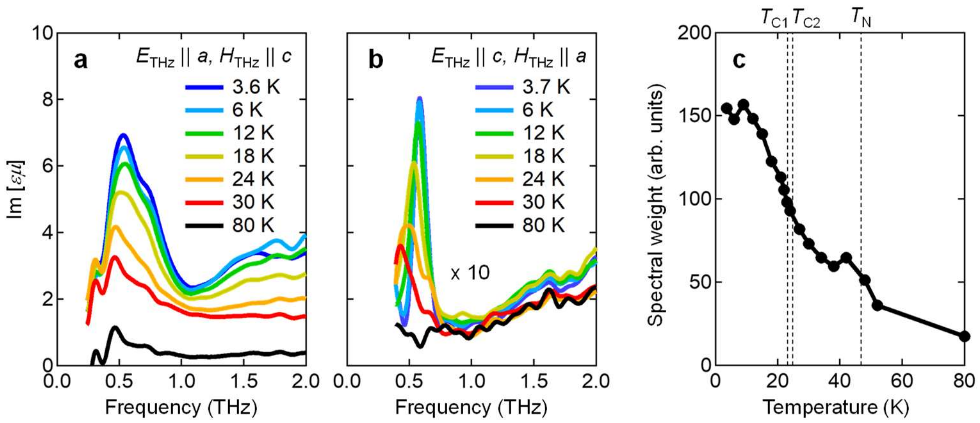

Supplementary Figure 2| Temperature dependence of terahertz spectra for EYMO. **a**, The electromagnon with $E_{THz}||a$. **b,** the AFMR with $H_{THz}||a$. **c**, Temperature dependence of spectral wight of electromagnon. All data of EYMO is reproduced from ref [5]. Here the spectra are plotted by effective dielectric constant εμ, which includes both electric and magnetic responses. To compare the intensity of terahertz spectra with that for electronic excitations in Fig. 1b, we plot the optical conductivity defined as $\sigma_1(\omega)=\varepsilon_0\mathrm{Im}[\varepsilon(\omega)\mu(\omega)]\omega$.

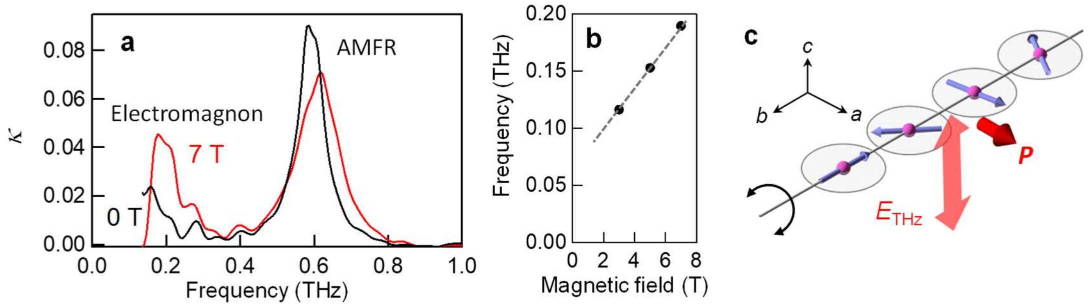

Supplementary Figure 3| Electromagnon induced by inverse Dzyaloshinskii–Moriya interaction. **a**, Terahertz spectra of EYMO at 4 K with $E_{THz}||c$, $H_{THz}||a$. A lower lying peak at 0.2 THz (7 T) is electromagnon and a higher lying peak at 0.6 THz is AFMR. **b**, The magnetic field dependence of the peak frequency of electromagnon. The peak frequency at zero magnetic field is estimated to be ~ 60 GHz. **c**, This lower energy electromagnon is ascribed to the rotational oscillation of spontaneous polarization, i.e., the rotation of spin spiral plane with rotation axis along the *b* axis[6,7]. In ab-plane cycloidal spin phase with $P||a$, the electromagnon has the transition electric dipole along the *c* axis. All data are reproduced from ref [7].

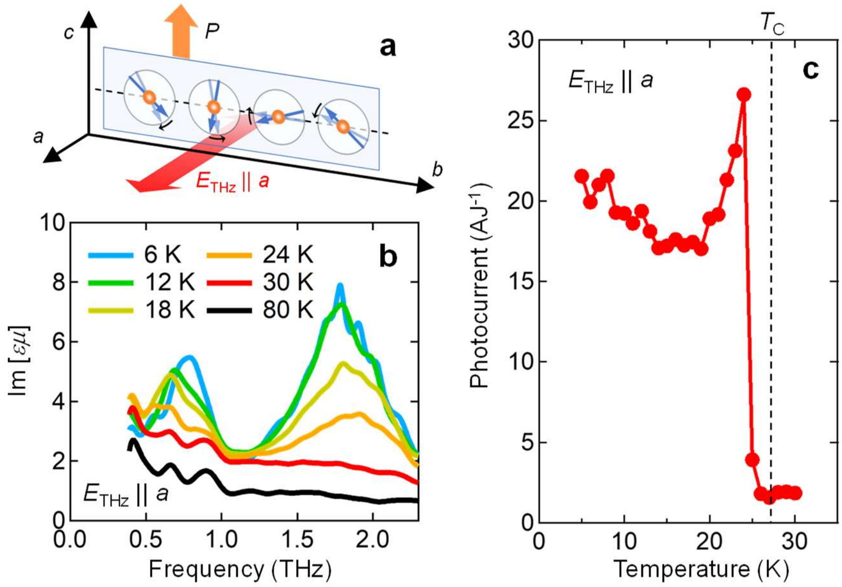

Supplementary Figure 4| Electromagnon and terahertz photocurrent for $TbMnO_3$ with *bc*-plane spin spiral. a, Schematics of *bc*-plane spin cycloid and electromagnon with $E_{THz}||a$. The *bc*-plane spin cycloid with spiral axis along the *b* axis induces the spontaneous *P* along the *c* axis (orange arrow), while the electromagnon addressed in this work always has the electric transition dipole along the *a* axis similar to the EYMO. **b**, Temperature dependence of electromagnon for $TbMnO_3$ partly reproduced from ref [3]. **c**, Temperature dependence of amplitude of photocurrent flowing along the *c* axis, which is parallel to the *P*. The enhancement of photocurrent is observed at low temperature and just below T_C (~28 K) similar to EYMO. The photocurrent is suppressed in the paraelectric phase (> T_C) as expected from the symmetry argument. Note that the amplitude of photocurrent for $TbMnO_3$ is much smaller than that for EYMO (see Fig. 3), and tiny residual signal discerned above T_C is due to experimental error.

References

1. Holstein, T. & Primakoff, H. Field Dependence of the Intrinsic Domain Magnetization of a Ferromagnet. *Phys. Rev.* **58**, 1098 (1940).
2. Morimoto, T., Kitamura, S. & Okumura. S., Electric polarization and nonlinear optical effects in noncentrosymmetric magnets. *Phys. Rev. B* **104,** 075139 (2021).
3. Takahashi, Y., et al. Evidence for an electric-dipole active continuum band of spin excitations in multiferroic $TbMnO_3$. *Phys. Rev. Lett.* **101**, 187201 (2008).
4. Hemberger, J., et al. Multiferroic phases of $Eu_{1-x}Y_xMnO_3$. *Phys. Rev. B* **75**, 035118 (2007).
5. Takahashi, Y., et al. Far-infrared optical study of electromagnons and their coupling to optical phonons in $Eu_{1-x}Y_xMnO_3$ ($x = 0.1, 0.2, 0.3, 0.4$, and 0.45). *Phys. Rev. B* **79**, 214431 (2009).
6. Katsura, H., Balatsky, A. V. & Nagaosa, N. Dynamical Magnetoelectric Coupling in Helical Magnets. *Phys. Rev. Lett.* **98**, 027203 (2007).
7. Takahashi, Y., Shimano, R., Kaneko, Y., Murakawa, H. & Tokura, Y. Magnetoelectric resonance with electromagnons in a perovskite helimagnet. *Nat. Phys.* **8**, 121 (2012).
